# Complete genome sequence of *Alicyclobacillus acidocaldarius* type strain (104-IA^T^)

**DOI:** 10.4056/sigs.591104

**Published:** 2010-01-28

**Authors:** Konstantinos Mavromatis, Johannes Sikorski, Alla Lapidus, Tijana Glavina Del Rio, Alex Copeland, Hope Tice, Jan-Fang Cheng, Susan Lucas, Feng Chen, Matt Nolan, David Bruce, Lynne Goodwin, Sam Pitluck, Natalia Ivanova, Galina Ovchinnikova, Amrita Pati, Amy Chen, Krishna Palaniappan, Miriam Land, Loren Hauser, Yun-Juan Chang, Cynthia D. Jeffries, Patrick Chain, Linda Meincke, David Sims, Olga Chertkov, Cliff Han, Thomas Brettin, John C. Detter, Claudia Wahrenburg, Manfred Rohde, Rüdiger Pukall, Markus Göker, Jim Bristow, Jonathan A. Eisen, Victor Markowitz, Philip Hugenholtz, Hans-Peter Klenk, Nikos C. Kyrpides

**Affiliations:** 1 DOE Joint Genome Institute, Walnut Creek, California, USA; 2 DSMZ – German Collection of Microorganisms and Cell Cultures GmbH, Braunschweig, Germany; 3 Los Alamos National Laboratory, Bioscience Division, Los Alamos, New Mexico, USA; 4 Biological Data Management and Technology Center, Lawrence Berkeley National Laboratory, Berkeley, California, USA; 5 Oak Ridge National Laboratory, Oak Ridge, Tennessee, USA; 6 HZI – Helmholtz Center for Infection Research, Braunschweig, Germany; 7 University of California Davis Genome Center, Davis, California, USA

**Keywords:** thermophile, acidophilic, aerobic, non-pathogenic, food spoilage, non-motile but encodes flagellar genes, GEBA

## Abstract

*Alicyclobacillus acidocaldarius* (Darland and Brock 1971) is the type species of the larger of the two genera in the bacillal family ‘*Alicyclobacillaceae*’. *A. acidocaldarius* is a free-living and non-pathogenic organism, but may also be associated with food and fruit spoilage. Due to its acidophilic nature, several enzymes from this species have since long been subjected to detailed molecular and biochemical studies. Here we describe the features of this organism, together with the complete genome sequence and annotation. This is the first completed genome sequence of the family ‘*Alicyclobacillaceae*’. The 3,205,686 bp long genome (chromosome and three plasmids) with its 3,153 protein-coding and 82 RNA genes is part of the *** G****enomic **** E****ncyclopedia of **** B****acteria and **** A****rchaea * project.

## Introduction

Strain 104-IA^T^ (= DSM 446 = ATCC 27009 = JCM 5260 = NCIMB 11725) is the type strain of the species *Alicyclobacillus acidocaldarius*, which is the type species of the genus *Alicyclobacillus* [1]. The genus currently consists of 20 species and two subspecies. Strain 104-IA^T^ was originally isolated as ‘*Bacillus acidocaldarius*’ in 1971 (or earlier) from a hot and acidic spring in Yellowstone National Park, USA. In 1992, it was reclassified on the basis of comparative 16S rRNA gene sequence analysis into the new genus *Alicyclobacillus* [1]. With the description of *A. acidocaldarius* subsp. *rittmannii* in 2002 [2] the subspecies name *A. acidocaldarius* subsp. *acidocaldarius* was automatically created following rule 46 of the bacteriological code [3], with 104-IAT as its type strain. (hereinafter nevertheless referred to as *A. acidocaldarius*, without subspecies epithet). The species name derives from ‘acidus’ from Latin meaning acidic combined with ‘caldarius’, Latin for ‘belonging to the hot’. Due to its thermoacidic nature, this species serves as a model organism for molecular and biochemical studies of its enzymes [4-19]. Strain 104-IA^T^ has also been used to produce the restriction enzyme BacI [20]. Here we present a summary classification and a set of features for *A. acidocaldarius* 104-IA^T^, together with the description of the complete genomic sequencing and annotation.

## Classification and features

The type strain 104-IA^T^ and several other strains were isolated from acidic hot springs in the Yellowstone National Park, USA, from soil from an acid fumarole in the Hawaiian Volcano National Park [21], and also from acidic environments in Japan [22]. Other strains, as identified by 16S rRNA gene sequences and by metabolic traits, were isolated from orchard soil, mango juice, vinegar flies or pre-pasteurized pear puree in South Africa [23-25]. These findings are supported by the experimentally determined heat resistance of *A. acidocaldarius* strains in water, acidic buffer and orange juice [26]. Thus, *A. acidocaldarius* might be involved in food and fruit spoilage, which is a characteristic of several other species of the genus *Alicyclobacillus* [23-25,27]. Clones with high sequence similarity (99%, AB042056) with the 16S rRNA gene sequence of strain 104-IA^T^ are reported by the NCBI BLAST server from a ‘simulated low level waste site’ in USA (GQ263212), but not with any metagenomic environmental samples (October 2009).

**Figure 1:**
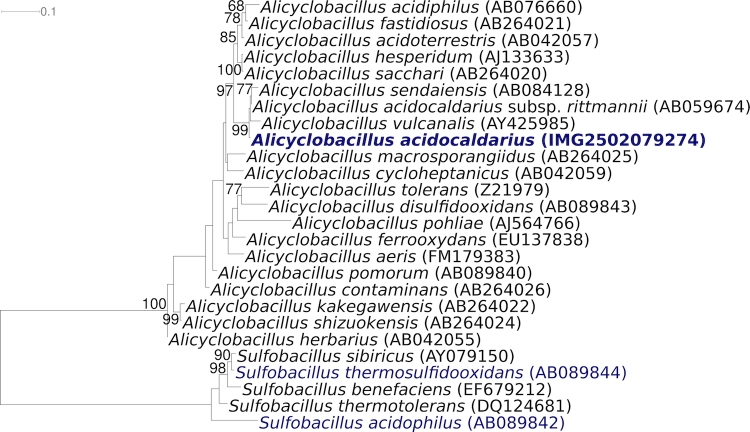
Phylogenetic tree highlighting the position of *A. acidocaldarius* 104-IA^T^ relative to the other type strains within the family. The tree was inferred from 1,419 aligned characters [28,29] of the 16S rRNA gene sequence under the maximum likelihood criterion [30] and rooted with the genus *Sulfobacillus*. The branches are scaled in terms of the expected number of substitutions per site. Numbers above branches are support values from 1,000 bootstrap replicates if larger than 60%. Lineages with type strain genome sequencing projects registered in GOLD [31] are shown in blue, published genomes in bold.

Figure 1 shows the phylogenetic neighborhood of for *A. acidocaldarius* 104-IA^T^ in a 16S rRNA based tree. The sequences of the six 16S rRNA gene copies in the genome of *A. acidocaldarius* 104-IA^T^, differ from each other by up to six nucleotides, and differ by up to five nucleotides from the previously published 16S rRNA sequence derived from DSM 446 (AJ496806).

**Figure 2:**
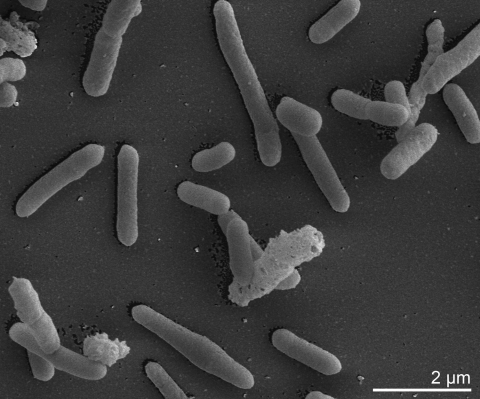
Scanning electron micrograph of *A. acidocaldarius* 104-IA^T^

On *B. acidocaldarius* medium (BAM medium) [32] strain 104-IA^T^ forms round, slightly mucous, creamy-white colonies after 72 hours of growth with a diameter of 1-4 mm and rod shaped cells that were 2.0-4.5 μm long and 0.5-1.0 μm wide (Table 1 and Figure 2) [22]. The endospores are terminal or subterminal and the sporangia are not swollen [22]. The upper and lower pH growth limits are pH 2 and pH 6 [21]. Strain 104-IA^T^ grows on basal medium supplemented with glucose, galactose, casamino acids, starch, glycerol, sucrose, gluconate, inositol, ribose, rhamnose, and lactose, but not with ethanol, sorbitol, sodium acetate, succinic acid, and sodium citrate [21]. Strain 104-IA^T^ hydrolyses gelatin and starch but is oxidase negative and does not reduce nitrate to nitrite [43]. Strain 104-IA^T^ produces acid from glycerol, L-arabinose, D-xylose, D-galactose, rhamnose, mannitol, methyl-α-D-glucoside, arbutin, aesculin, salicin, cellobiose, maltose, lactose, melibiose, sucrose, trehalose, D-raffinose, starch, and glycogen, but it does not produce acid from erythritol, D-arabinose, L-xylose, L-sorbose, inositol, sorbitol, methyl-α-D-mannisode, amygdalin, melezitose, xylitol, β-gentibiose, D-turanose, D-lyxose, D-tagatose, D-fucose, and 5-ketogluconate [43]. These acid production characteristics are largely congruent with the results from [22], however, L-sorbose, salicin, D-raffinose, starch, and D-turanose deviate across the studies [22,43].

**Table 1:** Classification and general features of *A. acidocaldarius* 104-IA^T^ in accordance with the MIGS recommendations [33]

**MIGS ID**	**Property**	**Term**	**Evidence code**
	Current classification	Domain *Bacteria*	TAS [34]
		Phylum *‘Firmicutes’*	TAS [35-37]
		Class *Bacilli*	TAS [36]
		Order *Bacillales*	TAS [38,39]
		Family ‘*Alicyclobacillaceae’*	TAS [40]
		Genus *Alicyclobacillus*	TAS [1]
		Species *Alicyclobacillus acidocaldarius*	TAS [21]
		Type strain 104-IA	TAS [21]
	Gram stain	positive	TAS [21]
	Cell shape	small rods	TAS [1]
	Motility	not reported (relevant genes missing)	NAS
	Sporulation	refractile endospores	TAS [21]
	Temperature range	45°C-70°C	TAS [21]
	Optimum temperature	60°C-65°C	TAS [21]
	Salinity	does not grow with 5% (w/v) NaCl	TAS [22]
MIGS-22	Oxygen requirement	strictly aerobic	TAS [21]
	Carbon source	saccharolytic	TAS [21]
	Energy source	carbohydrates	TAS [21]
MIGS-6	Habitat	hot acidic springs and soil	TAS [21]
MIGS-15	Biotic relationship	free living	NAS
MIGS-14	Pathogenicity	none	NAS
	Biosafety level	1	TAS [41]
	Isolation	acid hot spring	TAS [21]
MIGS-4	Geographic location	Nymph Creek, Yellowstone National Park, USA	TAS [21]
MIGS-5	Sample collection time	about 1970	TAS [21]
MIGS-4.1	Latitude	44.376	NAS
MIGS-4.2	Longitude	110.690	NAS
MIGS-4.3	Depth	not reported	
MIGS-4.4	Altitude	not reported	

Evidence codes - IDA: Inferred from Direct Assay (first time in publication); TAS: Traceable Author Statement (i.e., a direct report exists in the literature); NAS: Non-traceable Author Statement (i.e., not directly observed for the living, isolated sample, but based on a generally accepted property for the species, or anecdotal evidence). These evidence codes are from of the Gene Ontology project [42]. If the evidence code is IDA, then the property was directly observed for a living isolate by one of the authors or an expert mentioned in the acknowledgements.

Motility has not been reported for strain 104-IA^T^, although closely related species from the genus *Alicyclobacillus* are motile [22,27,43-45], which suggests a recent loss of motility in *A. acidocaldarius*. Indeed, strain 104-IA^T^ appears to have all genes necessary for a flagellum. However, essential genes for type 3 secretion system chaperones (*flgN*, *fliJ*, *fliT*) and for flagellar gene expression (*flhC*, *flhD*) are missing in the genome, which finally explains the non-motile phenotype.

### Chemotaxonomy

Characteristic for several *Alicyclobacillus* species is the presence of a large amount of ω-alicyclic fatty acids [1,46]. As such, strain 104-IA^T^ has approximately 51 ω-cyclohexane C_17:0_ and 33% ω-cyclohexane C_19:0_. Other fatty acids such as C_16:0_, C_18:0_, iso-C_15:0_, iso-C_16:0_, iso-C_18:0_, anteiso-C_15:0_, and anteiso-C_17:0_ amount at individual levels of approximately 1% to 5% [22,43]. Fatty acid composition is rather stable though not static across different temperature and pH values [47]. Moreover, strain 104-IA^T^ produces hopanoids, a group of pentacyclic triterpenoids, which together with the fatty acids constitute the lipophilic core of the cytoplasmic membrane. The amount of hopanoids depends on the temperature more so than the pH value [48]. The main isoprenoid quinone is menaquinone with seven isoprene units (MK-7) [1].

## Genome sequencing and annotation

### Genome project history

This organism was selected for sequencing on the basis of its phylogenetic position, and is part of the *** G****enomic **** E****ncyclopedia of **** B****acteria and **** A****rchaea * project. The genome project is deposited in the Genome OnLine Database [31] and the complete genome sequence is deposited in GenBank. Sequencing, finishing and annotation were performed by the DOE Joint Genome Institute (JGI). A summary of the project information is shown in Table 2.

**Table 2:** Genome sequencing project information

**MIGS ID**	**Property**	**Term**
MIGS-31	Finishing quality	Finished
MIGS-28	Libraries used	One Sanger 8 kb pMCL200 library and one 454 pyrosequencing standard library
MIGS-29	Sequencing platforms	ABI3730, 454 GS FLX
MIGS-31.2	Sequencing coverage	7.0 ×Sanger; 27.3× pyrosequencing
MIGS-30	Assemblers	Newbler, Phrap
MIGS-32	Gene calling method	Prodigal, GenePRIMP
	INSDC ID	CP001727 (chromosome) CP001728-30 (plasmids)
	GenBank Date of Release	September 10-14, 2009
	GOLD ID	Gc01110
	NCBI project ID	29405
	Database: IMG-GEBA	2501939636
MIGS-13	Source material identifier	DSM 446
	Project relevance	Tree of Life, GEBA

### Growth conditions and DNA isolation

*A. acidocaldarius* 104-IA^T^, DSM 446, was grown in DSM Medium 402 [49] at 60°C. DNA was isolated from 0.5-1 g of cell paste using Qiagen Genomic 500 DNA Kit (Qiagen, Hilden, Germany) with cell lysis modification st/L [50] and one hour incubation at 37°C.

### Genome sequencing and assembly

The genome was sequenced using a combination of Sanger and 454 sequencing platforms. All general aspects of library construction and sequencing performed at the JGI can be found at http://www.jgi.doe.gov/. 454 Pyrosequencing reads were assembled using the Newbler assembler version 1.1.02.15 (Roche). Large Newbler contigs were broken into 3,478 overlapping fragments of 1,000 bp and entered into assembly as pseudo-reads. The sequences were assigned quality scores based on Newbler consensus q-scores with modifications to account for overlap redundancy and to adjust inflated q-scores. A hybrid 454/Sanger assembly was made using the parallel phrap assembler (High Performance Software, LLC). Possible mis-assemblies were corrected with Dupfinisher [51] or transposon bombing of bridging clones (Epicentre Biotechnologies, Madison, WI). Gaps between contigs were closed by editing in Consed, custom primer walk or PCR amplification. A total of 767 Sanger finishing reads were produced to close gaps, to resolve repetitive regions, and to raise the quality of the finished sequence. The error rate of the completed genome sequence is less than 1 in 100,000. The final assembly contains 24,980 Sanger and 363,136 Pyrosequencing reads. Together all sequence types provided 34.3 × coverage of the genome

### Genome annotation

Genes were identified using Prodigal [52] as part of the Oak Ridge National Laboratory genome annotation pipeline, followed by a round of manual curation using the JGI GenePRIMP pipeline [53]. The predicted CDSs were translated and used to search the National Center for Biotechnology Information (NCBI) nonredundant database, UniProt, TIGRFam, Pfam, PRIAM, KEGG, COG, and InterPro databases. Additional gene prediction analysis and manual functional annotation were performed within the Integrated Microbial Genomes Expert Review (IMG-ER) platform [54].

## Genome properties

**Figure 3:**
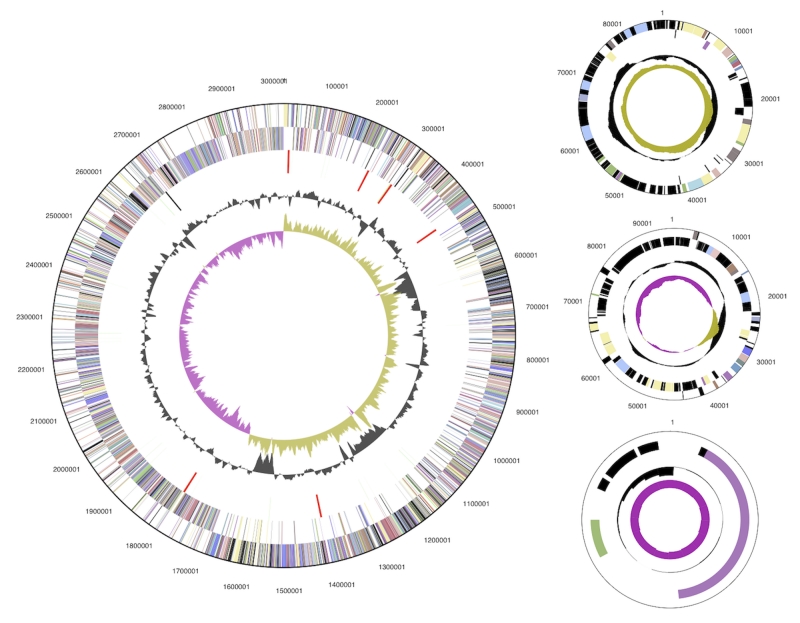
Graphical circular map of the chromosome and plasmids. From outside to the center: Genes on forward strand (color by COG categories), Genes on reverse strand (color by COG categories), RNA genes (tRNAs green, rRNAs red, other RNAs black), GC content, GC skew.

The genome consists of a 3,018,755 bp long chromosome and three plasmids of 91,726 bp, 87,298 bp, and 7,907 bp (Table 3 and Figure 3). Of the 3,235 genes predicted, 3,153 were protein-coding genes, and 82 RNAs; 69 pseudogenes were also identified. The majority of the protein-coding genes (68.4%) were assigned with a putative function while those remaining were annotated as hypothetical proteins. The distribution of genes into COGs functional categories is presented in Table 4.

**Table 3:** Genome Statistics

**Attribute**	**Value**	**% of Total**
Genome size (bp)	3,205,686	100.00%
DNA coding region (bp)	2,907,874	90.71%
DNA G+C content (bp)	1,984,066	61.89%
Number of replicons	4	
Extrachromosomal elements	3	
Total genes	3,235	100.00%
RNA genes	82	2.53%
rRNA operons	6	
Protein-coding genes	3,153	97.47%
Pseudo genes	82	2.13%
Genes with function prediction	2,214	68,44%
Genes in paralog clusters	661	20.43%
Genes assigned to COGs	2,221	68.66%
Genes assigned Pfam domains	2,297	71.00%
Genes with signal peptides	686	21.21%
Genes with transmembrane helices	858	26.52%
CRISPR repeats	4	

**Table 4:** Number of genes associated with the general COG functional categories

**Code**	**value**	**%age**	**Description**
J	147	4.7	Translation, ribosomal structure and biogenesis
A	0	0.0	RNA processing and modification
K	191	6.1	Transcription
L	177	5.6	Replication, recombination and repair
B	0	0.0	Chromatin structure and dynamics
D	33	1.0	Cell cycle control, mitosis and meiosis
Y	0	0.0	Nuclear structure
V	29	0.9	Defense mechanisms
T	110	3.5	Signal transduction mechanisms
M	124	3.9	Cell wall/membrane biogenesis
N	58	1.8	Cell motility
Z	0	0.0	Cytoskeleton
W	0	0.0	Extracellular structures
U	59	1.9	Intracellular trafficking and secretion
O	78	2.5	Posttranslational modification, protein turnover, chaperones
C	130	4.1	Energy production and conversion
G	203	6.4	Carbohydrate transport and metabolism
E	201	6.4	Amino acid transport and metabolism
F	61	1.9	Nucleotide transport and metabolism
H	117	3.7	Coenzyme transport and metabolism
I	120	3.8	Lipid transport and metabolism
P	104	3.3	Inorganic ion transport and metabolism
Q	58	1.8	Secondary metabolites biosynthesis, transport and catabolism
R	266	8.4	General function prediction only
S	185	5.9	Function unknown
-	1014	32.2	Not in COGs
